# Ethanol and High Cholesterol Diet Causes Severe Steatohepatitis and Early Liver Fibrosis in Mice

**DOI:** 10.1371/journal.pone.0163342

**Published:** 2016-09-27

**Authors:** Yasodha Krishnasamy, Venkat K. Ramshesh, Monika Gooz, Rick G. Schnellmann, John J. Lemasters, Zhi Zhong

**Affiliations:** 1 Department of Drug Discovery & Biomedical Sciences, Medical University of South Carolina, Charleston, South Carolina, United States of America; 2 Ralph H. Johnson Veterans Affairs Medical Center, Charleston, South Carolina, United States of America; 3 Department of Biochemistry & Molecular Biology, Medical University of South Carolina, Charleston, South Carolina, United States of America; 4 Institute of Theoretical & Experimental Biophysics, Russian Academy of Sciences, Pushchino, Moscow Region, Russian Federation; The University of Hong Kong, HONG KONG

## Abstract

**Background and Aim:**

Because ethanol consumption is commonly associated with a high cholesterol diet, we examined whether combined consumption of ethanol and high cholesterol increases liver injury and fibrosis.

**Methods:**

Male C57BL/6J mice were fed diets containing: 1) 35% of calories from corn oil (CTR), 2) CTR plus 0.5% (w/v) cholesterol (Chol), 3) CTR plus ethanol (27% of calories) (EtOH), or 4) EtOH+Chol for 3 months.

**Results:**

In mice fed Chol or EtOH alone, ALT increased to ~160 U/L, moderate hepatic steatosis occurred, and leukocyte infiltration, necrosis, and apoptosis increased modestly, but no observable fibrosis developed. By contrast in mice fed EtOH+Chol, ALT increased to ~270 U/L, steatosis was more extensive and mostly macrovesicular, and expression of proinflammatory molecules (HMGB-1, TLR4, TNFα, ICAM-1) and leukocyte infiltration increased substantially. Necrosis and apoptosis also increased. Trichrome staining and second harmonic generation microscopy revealed hepatic fibrosis. Fibrosis was mostly sinusoidal and/or perivenular, but in some mice bridging fibrosis occurred. Expression of smooth muscle α-actin and TGF-β1 increased slightly by Chol, moderately by EtOH, and markedly by EtOH+Chol. TGF-β pseudoreceptor BAMBI increased slightly by Chol, remained unchanged by EtOH and decreased by EtOH+Chol. MicroRNA-33a, which enhances TGF-β fibrotic effects, and phospho-Smad2/3, the down-stream signal of TGF-β, also increased more greatly by EtOH+Chol than Chol or EtOH. Metalloproteinase-2 and -9 were decreased only by EtOH+Chol.

**Conclusion:**

High dietary cholesterol and chronic ethanol consumption synergistically increase liver injury, inflammation, and profibrotic responses and suppress antifibrotic responses, leading to severe steatohepatitis and early fibrosis in mice.

## Introduction

Alcoholic liver disease (ALD) affects more than 2.5 million people in U.S [1,2]. The pathological changes of human ALD are characterized in three major categories: steatosis, hepatitis, and fibrosis/cirrhosis, the last of which ultimately leads to end-stage liver disease and frequently liver cancer [1,2]. These three pathological types can exist independently but often co-exist. ALD accounts for ~50% of deaths due to cirrhosis and ~30% of all liver disease-related deaths in the U.S [3–7]. Death is also a frequent outcome when inflammation of the liver occurs [8,9]. Cirrhosis superimposed with alcoholic hepatitis results in a death rate of more than 60% over 4-years [10]. Despite extensive studies, mechanisms by which ethanol damages the liver are far from clear, and effective treatment for ALD is lacking. For patients with cirrhosis and liver failure, nutrition and supportive care are the major therapies. However, these treatments at best only delay progression of ALD.

ALD is frequently the result of heavy drinking. However, other co-existing risk factors markedly increase liver injury and fibrosis [2,10]. Gender, chronic viral hepatitis, HIV infection, hemochromatosis, genetic factors, obesity, and smoking all affect the progression of ALD. Nutritional factors also appear to play an important role in ethanol-induced liver injury and fibrosis/cirrhosis [10,11], and alcohol in combination with malnutrition increases ALD in human and in animals [12,13]. Fish oil and corn oil promote whereas beef fat decreases ethanol-induced liver injury [11,14].

Epidemiological studies show that high cholesterol intake is associated with increased risk of liver fibrosis and cancer [15]. Some recent reports indicate that high cholesterol intake exacerbates liver fibrosis after bile duct ligation, carbon tetrachloride treatment, methionine-choline deficiency (3 months) and high fat diet (6 months) in mice [16,17]. Since ethanol consumption and high cholesterol intake frequently co-exist in Western diets, we examined the combined effects of chronic ethanol and high cholesterol intake on liver injury and fibrosis.

## Methods

### Animals

Male C57BL/6J mice (8–10 weeks) from Jackson Laboratory were fed one of four modified Lieber-DeCarli liquid diets (Dyets, Bethleham, PA): 1) a control liquid (CTR) diet with 35% calories from corn oil and 27% of calories from maltose dextrin (Dyets), 2) a high cholesterol diet (Chol) with 0.5% (W/V) cholesterol (Dyets) added to CTR, 3) an ethanol (EtOH) diet with 35% calories from corn oil and 27% of calories from ethanol, and 4) an ethanol plus cholesterol (EtOH+Chol) diet with 35% calories from corn oil, 27% of calories from ethanol and 0.5% (W/V) cholesterol. Concentrations of ethanol in liquid diets were increased stepwise (9% of calories every 2 days), and mice were then fed the liquid diets for 3 months after ethanol reached 27% of calories. In preliminary studies, we found that Chol did not alter the volume of diet consumption, whereas EtOH decreased diet consumption. Therefore, daily consumption of ethanol-containing liquid diets (EtOH and EtOH+Chol) were measured, and the same volumes were given to the CTR and Chol groups on the following day (pair-feeding) to balance caloric intake.

### Measurement of serum alanine aminotransferase (ALT)

After 3 months of liquid diet feeding, the abdomen was opened under pentobarbital anesthesia (80 mg/kg, *i*.*p*.), and blood was collected from inferior vena cava. Serum was obtained by centrifugation and kept at -80°C. Alanine transaminase (ALT) was measured using a kit from Pointe Scientific (Canton, MI).

### Measurement of hepatic triglycerides, total cholesterol, and hydroxyproline

Hepatic triglycerides in liver homogenates were extracted with 2:1 chloroform/methanol and measured using an analytical kit (Enzymatic Standbio, Boerne, TX), as described previously [18]. After hydrolyzing liver tissue in sodium hydroxide (2N) at 120°C, hydroxyproline was detected as described elsewhere and expressed as μg/g liver wet weight [19].

To measure cholesterol, liver tissue (10 mg) was homogenized in 200 μL of a mixture of chloroform:isopropanol:NP-40 (7:11:0.1) and then centrifuged at 15,000X g for 10 min at room temperature. The supernatant (organic phase) was transferred to a new tube, dried in air at 50°C on a hotplate until the solvents were removed, and then kept under vacuum at room temperature for 30 min to remove trace amounts of solvents. The resulting lipid pellets were dissolved in 200 μl of 1x assay diluent from the Total Cholesterol Assay Kit (Cell Biolabs Inc, San Diego, CA). Cholesterol in 50 μl of samples was measured using the assay kit according to the manufacturer’s protocol.

### Histology and detection of fibrosis on liver sections

Under pentobarbital anesthesia as described above, the liver was infused with ~2 mL normal saline and then harvested. Liver tissue was fixed in 4% paraformaldehyde in phosphate buffer for 48 h and processed for paraffin sections [20]. Liver sections were stained with hematoxylin and eosin (H&E), and images were captured under a microscope (Zeiss Axiovert 100 microscope, Thornwood, NY) using a 20x objective lens.

Some liver slides were processed at the Histological Core of Medical University of South Carolina and stained with the Mason’s Trichrome staining to reveal liver fibrosis. Images were captured using a Zeiss Axiovert 100 microscope (Thornwood, NY) and a 10x objective lens [20].

Additionally, liver fibrosis was detected by second harmonic generation (SHG) microscopy of liver sections. When intense laser light passes through a material with a non-linear molecular structure such as collagen, the interaction of laser light with the nonlinear material generates second-harmonic light which possesses twice the energy but half the wavelength of the original light [21]. Therefore, collagen fibers can be imaged by SHG without special staining or fluorophores. On de-paraffinized, unstained slides, SHG imaging was performed using an Olympus FluoView 1200MPE laser scanning multiphoton microscope (Olympus, Center Valley, PA) and a 30x 1.2 N.A. water-immersion objective lens. Second harmonic imaging was performed with 900-nm light. The emission wavelength was 450 nm [22].

### Immunoblotting

After liver harvest under pentobarbital anesthesia, liver tissue was snap-frozen in liquid nitrogen and kept at -80°C until use. Proteins of interest were detected by immunoblotting, as described previously [20]. Membranes were blotted with primary antibodies against cleaved caspase-3 (CC3), fatty acid synthase (FAS), actin (Cell Signaling Technology, Danvers, MA), myeloperoxidase (MPO, DAKO Corp., Carpinteria, CA), CD4 (Origene Technologies, Rockville, MD), high mobility group box-1 (HMGB-1), toll-like receptor-4 (TLR4), Type I collagen, transforming growth factor-β1 (TGF-β1), cytochrome p450 2E1 (Cyp2E1) (Abcam, Cambridge, MA), 4-hydroxynonenal adducts (4-HNE, Alpha Diagnostic, San Antonio, TX), Smad2/3 and phospho-Smad2/3, bone morphogenic protein and activin membrane-bound inhibitor (BAMBI), alcohol dehydrogenase (ADH), aldehyde dehydrogenase-2 (ALDH2), carnitine palmitoyltranferase-1 (Cpt1) (Santa Cruz Biotech., Santa Cruz, CA), metalloproteinase-2 (MMP-2, Calbiochem, Billerica, MA), metalloproteinase-9 (MMP-9), intracellular adhesion molecule-1 (ICAM-1; BD Biosciences Pharmingen, San Diego, CA), F4/80 (Serotech, Raleigh, NC), and smooth muscle α-actin (α-SMA, DAKO, Carpinteria, CA) at concentrations of 1:1000 to 1:3000 overnight at 4°C. Horseradish peroxidase-conjugated secondary antibodies of appropriate species were then applied, and detection was by chemiluminescence (Pierce Biotechnology, Rockford, IL).

### Detection of the X-box binding protein 1 (XBP-1) mRNA by quantitative real-time PCR

The mRNA of XBP-1 was detected by RT-qPCR, as described elsewhere [20], using the following primers: forward: ACACGCTTGGGAATGGACAC and reverse: CCATGGGAAGATGTTCTGGG. The abundance of mRNAs was normalized against hypoxanthine phospho-ribosyl-transferase (HPRT) using the ΔΔ*Ct* method.

### Detection of miRNA 33a in liver tissue

Small RNA (<100 nucleotides) enriched in miRNA was extracted from liver tissue using High Pure^™^ miRNA isolation kit (Roche^®^, Indianapolis, IN) according to the manufacturer’s protocol. cDNAs were synthesized from 10 ng RNA using Universal cDNA synthesis Kit (Exiqon, Woburn, MA). Real-time PCR reaction was performed using a miRCURY^™^LNA Real-time PCR kit (Exiqon) and the Bio-rad CFX 96 Real time PCR System with incubation at 95°C for 10 min, followed by 40 cycles at 95°C for 15 seconds and 60°C for 1 min. The sequence of miRNA-33a probe is 5’-GUGCAUUGUAGUUGCAUUGCA-3’. The results were normalized to the expression of U6 miRNA (the reference gene, probe sequence: 5’-GTGCTCGCTTCGGCAGCACATATACTAAAATTGGAACGATACAGAGAAGATTAGCATGGCCCCTGCGCAAGGATGACACGCAAATTCGTGAAGCGTTCCATATTTT-3’). Differential expression was determined by the delta-delta Ct method.

### Statistical analysis

Data shown are means ± S.E.M. (4 mice per group). Groups were compared using ANOVA plus Student-Newman-Keuls posthoc test. Differences were considered significant at p<0.05.

### Ethics statement

All animals were given humane care in compliance with institutional guidelines using protocols approved by the Institutional Animal Care and Use Committee of the Medical University of South Carolina. All surgery was performed under sodium pentobarbital anesthesia (80 mg/kg, *i*.*p*.).

## Results

### Combined chronic ethanol consumption and high cholesterol intake increase liver steatosis and injury

**Fig 1:**
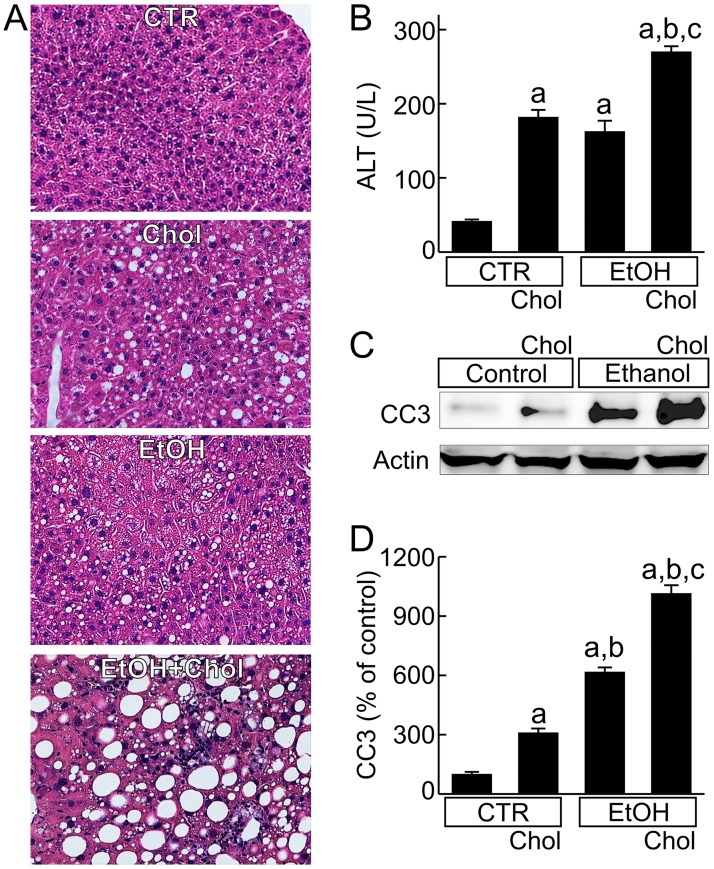
Combined ethanol and cholesterol feeding exacerbates liver injury and steatosis. Mice were fed CTR, Chol, EtOH or EtOH+Chol diets for 3 months. **A**, representative images from H&E stained liver sections. **B**, serum alanine aminotransferase (ALT). **C**, representative immunoblots of cleaved caspase-3 (CC3) and actin. **D**, quantification of CC3 immunoblots by densitometry. Values are means ± S.E.M. **a**, p < 0.05 vs CTR; **b**, p < 0.05 vs Chol; **c**, p < 0.05 vs EtOH (n = 4 per group).

In the livers from mice fed CTR, no pathological changes were observed, only some small fat droplets (Fig 1A). After feeding either Chol or EtOH, steatosis developed with mixed micro- and macrovesicular fat droplets. Steatosis was slightly greater in the Chol group than the EtOH group (Fig 1A). By contrast after EtOH+Chol feeding, much more marked steatosis developed with widespread formation of macrovesicular fat droplets that were larger in size and more abundant compared to Chol or EtOH (Fig 1A).

**Fig 2:**
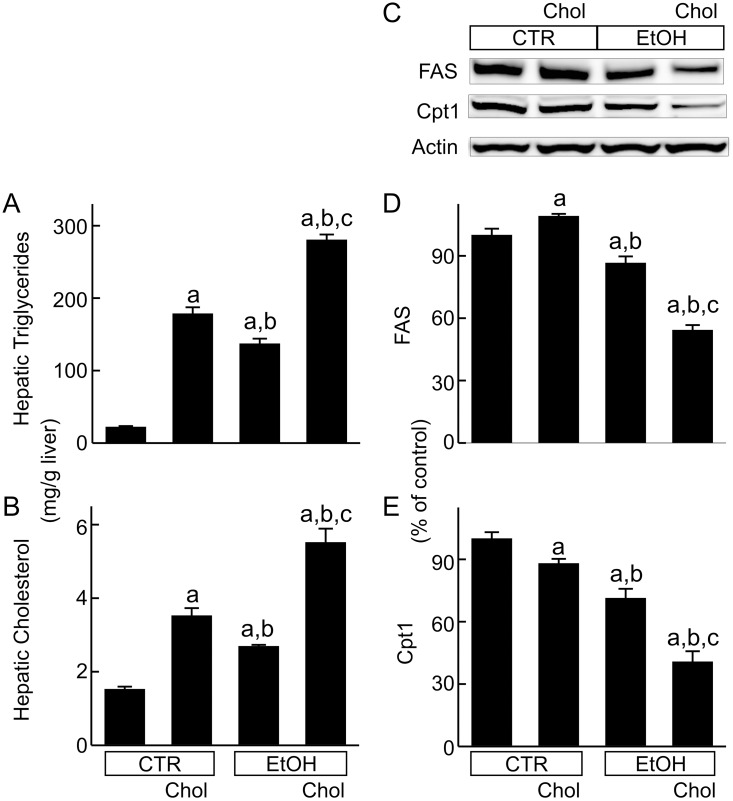
Combined ethanol and cholesterol feeding increases hepatic triglycerides and cholesterol but suppressed fatty acid synthase and carnitine palmitoyltransferase-1 expression. Mice were fed CTR, Chol, EtOH and EtOH+Chol diets for 3 months. Triglycerides (**A**) and cholesterol (**B**) in liver extracts were measured using commercial kits. FAS and Cpt1 were detected by immunoblotting (**C**, representative images). **D**, quantification of FAS immunoblots by densitometry. **E**, quantification of Cpt1 immunoblots. Values are means ± S.E.M. **a**, p < 0.05 vs CTR; **b**, p < 0.05 vs Chol; **c**, p < 0.05 vs EtOH (n = 3–4 per group).

Hepatic triglycerides increased 8-fold and 6-fold, respectively, after feeding Chol and EtOH diets (Fig 2A). By contrast after EtOH+Chol, triglycerides increased 13-fold (Fig 2A). Hepatic cholesterol increased 2.3-fold and 1.7-fold, respectively, with the Chol and EtOH diets (Fig 2B), whereas after feeding EtOH+Chol, hepatic cholesterol increased 3.7-fold (Fig 2B).

FAS, which catalyzes *de novo* fatty acid formation, increased slightly (9%) by Chol but decreased 13% by EtOH and 46% by EtOH+Chol (Fig 2C and 2D). Cpt1 catalyzes the transfer of the fatty acyl groups from coenzyme A (CoA) to carnitine to form palmitoylcarnitine, an essential step for transport of long chain fatty acids into mitochondria for β-oxidation [23]. After Chol and EtOH, respectively, Cpt1 decreased 12% and 29%, whereas after EtOH+Chol Cpt1 decreased 59% (Fig 2E).

Compared to CTR, serum ALT levels increased 4-fold after feeding Chol or EtOH. By contrast after feeding EtOH+Chol, serum ALT increased 6.4-fold (Fig 1B), indicating greater liver injury. Necrosis, apoptosis, and ballooning degeneration were observed in the livers from Chol-fed or EtOH-fed mice. These changes became more overt in livers from EtOH+Chol-fed mice (Fig 1A). Cleaved caspase-3, a marker of apoptosis, increased 3.1-fold by Chol, 6.1-fold by EtOH, and 10.1-fold by EtOH+Chol (Fig 1C and 1D).

### Combined chronic ethanol and high cholesterol intake synergistically increases inflammatory responses in the liver

**Fig 3:**
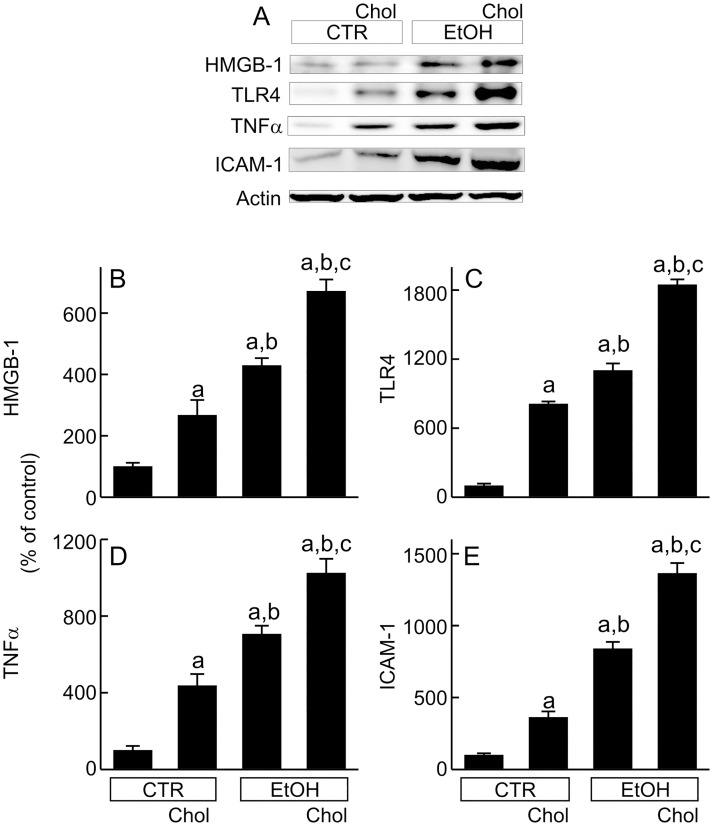
Combined ethanol and cholesterol feeding enhances inflammatory responses. Mice were fed CTR, Chol, EtOH and EtOH+Chol diets for 3 months. **A**, representative immunoblots of HMGB-1, TLR4, TNFα, ICAM-1 and actin. **B**, quantification of HMGB-1 immunoblots by densitometry. **C**, quantification of TLR4 immunoblots. **D**, quantification of TNFα immunoblots. **E**, quantification of ICAM-1 immunoblots. Values are means ± S.E.M. **a**, p < 0.05 vs CTR; **b**, p < 0.05 vs Chol; **c**, p < 0.05 vs EtOH (n = 4 per group).

HMGB-1 is up-regulated/released during cell stress and injury, acting as a damage-associated molecular pattern molecule (DAMP) that promotes inflammatory processes [24,25]. TLR4 (pattern recognition receptor [PRR] that initiates inflammatory responses), TNFα (cytotoxic and inflammatory cytokine), and ICAM-1 (adhesion molecule that mediates leukocyte adhesion) increased with Chol and EtOH feeding (Fig 3). Increases in these inflammatory molecules were larger after EtOH than Chol. However, EtOH+Chol feeding increased HMGB-1, TLR4, TNFα, and ICAM-1 to an even greater extent than Chol or EtOH alone (Fig 3).

**Fig 4:**
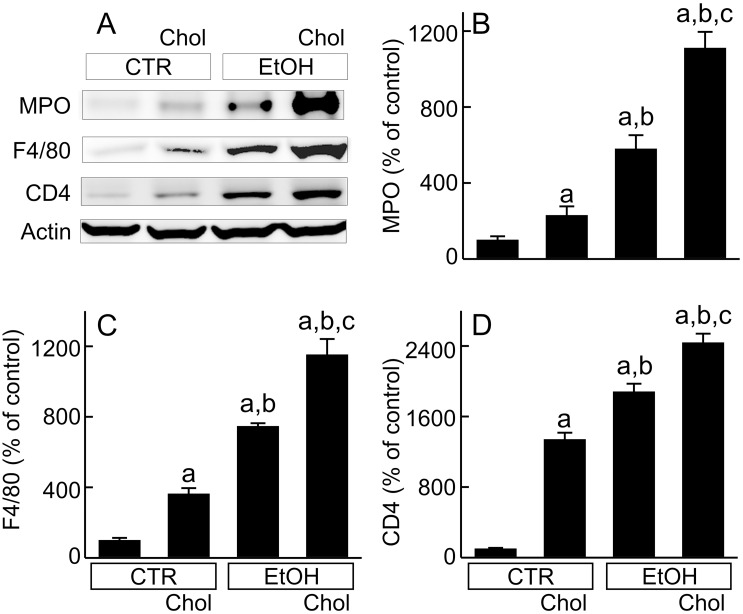
Combined ethanol and cholesterol feeding increases leukocyte infiltration in the liver. Mice were fed CTR, Chol, EtOH and EtOH+Chol diets for 3 months. **A**, representative immunoblots of MPO, F4/80, CD4 and actin. **B**, quantification of MPO immunoblots by densitometry. **C**, quantification of F4/80 immunoblots. **D**, quantification of CD4 immunoblots. Values are means ± S.E.M. **a**, p < 0.05 vs CTR; **b**, p < 0.05 vs Chol; **c**, p < 0.05 vs EtOH (n = 4 per group).

Histologically, leukocyte infiltration increased slightly after Chol and moderately after EtOH. However, leukocyte infiltration was even greater after EtOH+Chol (Fig 1A). MPO (marker of neutrophil infiltration), F4/80 (marker of monocytes/macrophages) and CD4 (marker of T lymphocytes) all increased after Chol and EtOH diets (Fig 4). EtOH caused more leukocyte infiltration than Chol. However, MPO, F4/80 and CD4 all increased to even greater levels in mice fed the EtOH+Chol diet (Fig 4).

### Combined chronic ethanol and cholesterol consumption causes liver fibrosis

**Fig 5:**
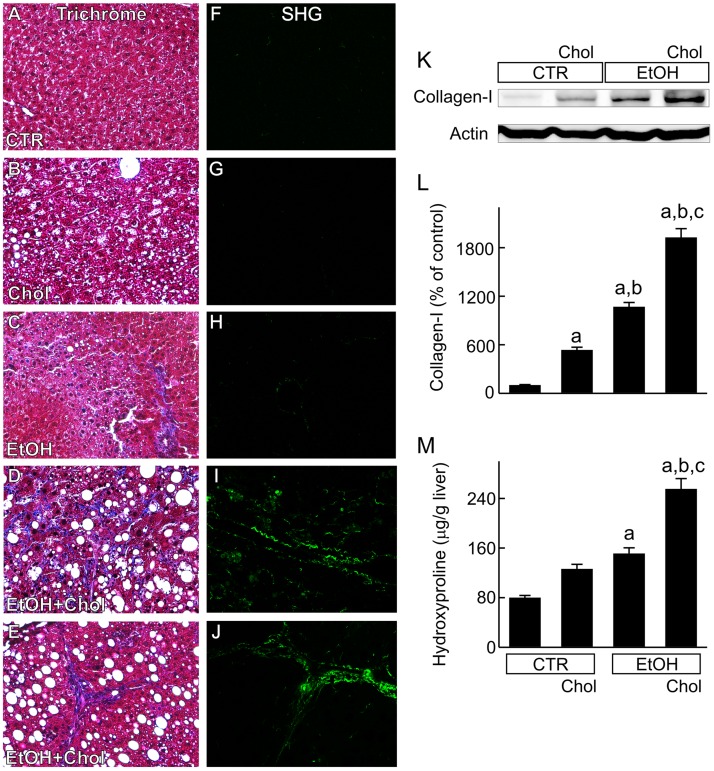
Combined ethanol and cholesterol feeding induces liver fibrosis and increases Type I collagen and hydroxyproline in the liver. Mice were fed CTR, Chol, EtOH and EtOH+Chol diets for 3 months. **A-E**, Mason’s Trichrome staining. **F-J**, second harmonic generation (SHG) microscopy. **K**, representative immunoblots of Type I collagen (Collagen-I) and actin. **L**, quantification of Type I collagen immunoblots by densitometry. **M**, hydroxyproline in the liver. Values are means ± S.E.M. **a**, p < 0.05 vs CTR; **b**, p < 0.05 vs Chol; **c**, p < 0.05 vs EtOH (n = 4 per group).

After Trichrome staining of liver sections from mice fed CTR and Chol diets, no fibrosis was detected (Fig 5A and 5B). Weak blue Trichrome staining appeared occasionally in some livers of mice fed EtOH (Fig 5C). However after EtOH+Chol, blue Trichrome staining increased in all livers. Fibrosis was predominantly sinusoidal and perivenular (Fig 5D), showing a “chicken-wire” pattern. Bridging fibrosis was present occasionally in some livers of EtOH+Chol-fed mice (Fig 5E).

Fibrosis was further verified by SHG microscopy. In livers of mice fed CTR, collagen SHG signals were barely detectable except around larger vessels (Fig 5F). Collagen SHG signals were unchanged in livers of Chol-fed mice compared to CTR mice (Fig 5G) but increased slightly in some liver from EtOH-fed mice (Fig 5H). By contrast, collagen SHG signals increased markedly in the livers from EtOH+Chol-fed mice. Most of collagen was located along sinusoids and in pericentral regions (Fig 5I), but bridging fibrosis was apparent in some mice (Fig 5J).

By Western blotting, Type I collagen, one of the collagens that form fibers in liver fibrosis, increased 5 folds in Chol-fed mice compared to CTR. In EtOH-fed mice, Type I collagen increased 10 folds. In EtOH+Chol-fed mice, Type I collagen increased to an even greater extent (19 folds) (Fig 5K and 5L).

Hepatic hydroxyproline, an indicator of collagen deposition, did not increase after Chol feeding but increased modestly after EtOH (Fig 5M). In mice fed EtOH+Chol, hydroxyproline increased more than after Chol and EtOH feeding (Fig 5M).

### Combined ethanol plus high cholesterol diet increases hepatic stellate cell activation and alters TGF-β signaling

**Fig 6:**
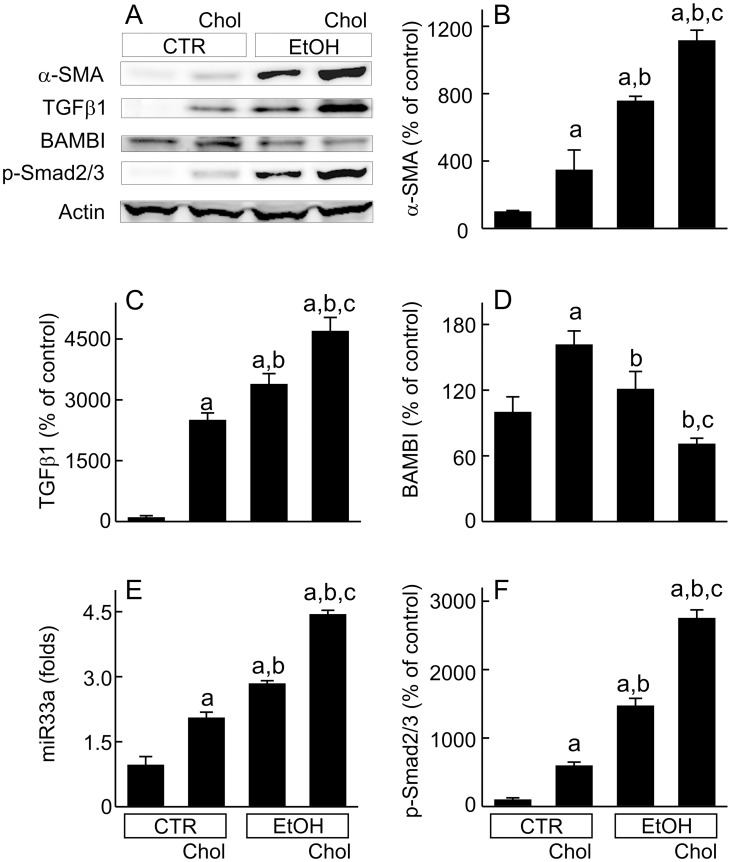
Combined ethanol and cholesterol feeding stimulates stellate cell activation and alters TGF-β signaling in the liver. Mice were fed CTR, Chol, EtOH and EtOH+Chol diets for 3 months. **A**, representative immunoblots of α-SMA, TGF-β1, BAMBI, Smad2/3, phospho-Smad2/3 and actin. **B**, quantification of α-SMA immunoblots by densitometry. **C**, quantification of TGF-β1 immunoblots. **D**, quantification of BAMBI immunoblots. **E**, detection of miR33a by RT-qPCR. **F**, quantification of phospho-Smad2/3 immunoblots. Values are means ± S.E.M. **a**, p < 0.05 vs CTR; **b**, p < 0.05 vs Chol; **c**, p < 0.05 vs EtOH (n = 4 per group).

Activated hepatic stellate cells (HSC) synthesize and excrete extracellular matrix (ECM), leading to liver fibrosis. Expression of α-SMA, a marker of HSC activation, was barely detectable in livers from mice fed CTR (Fig 6A). Hepatic α-SMA increased 3.5 folds in Chol-fed mice and 7.5 folds in EtOH-fed mice. After EtOH+Chol feeding, α-SMA increased to a greater extent than Chol alone or EtOH (11.2 folds) (Fig 6A and 6B).

TGF-β1 is a potent fibrogenic cytokine. TGF-β1 increased 25-fold by Chol feeding, 33-fold by EtOH feeding, and 47-fold by EtOH+Chol feeding (Fig 6A and 6C). BAMBI is a transmembrane TGF-β pseudoreceptor that silences TGF-β signaling [26,27]. BAMBI increased 60% in Chol-fed mice, remained unchanged in EtOH-fed mice, and decreased 30% in EtOH+Chol fed mice (Fig 6A and 6D). miR33a enhances TGF-β profibrogenic effects, possibly by modulating Smad signaling [28]. miR33a increased 2.0-, 2.8-, and 4.5-fold by Chol, EtOH, and EtOH+Chol feeding, respectively (Fig 6E). Smad2/3 expression was not altered in any groups (not shown). However, phospho-Smad2/3 increased 6-, 15-, and 28-fold by Chol, EtOH and EtOH+Chol feeding, respectively, indicating Smad2/3 activation (Fig 6A and 6F).

### Combined ethanol and cholesterol consumption decreases metalloproteinases

**Fig 7:**
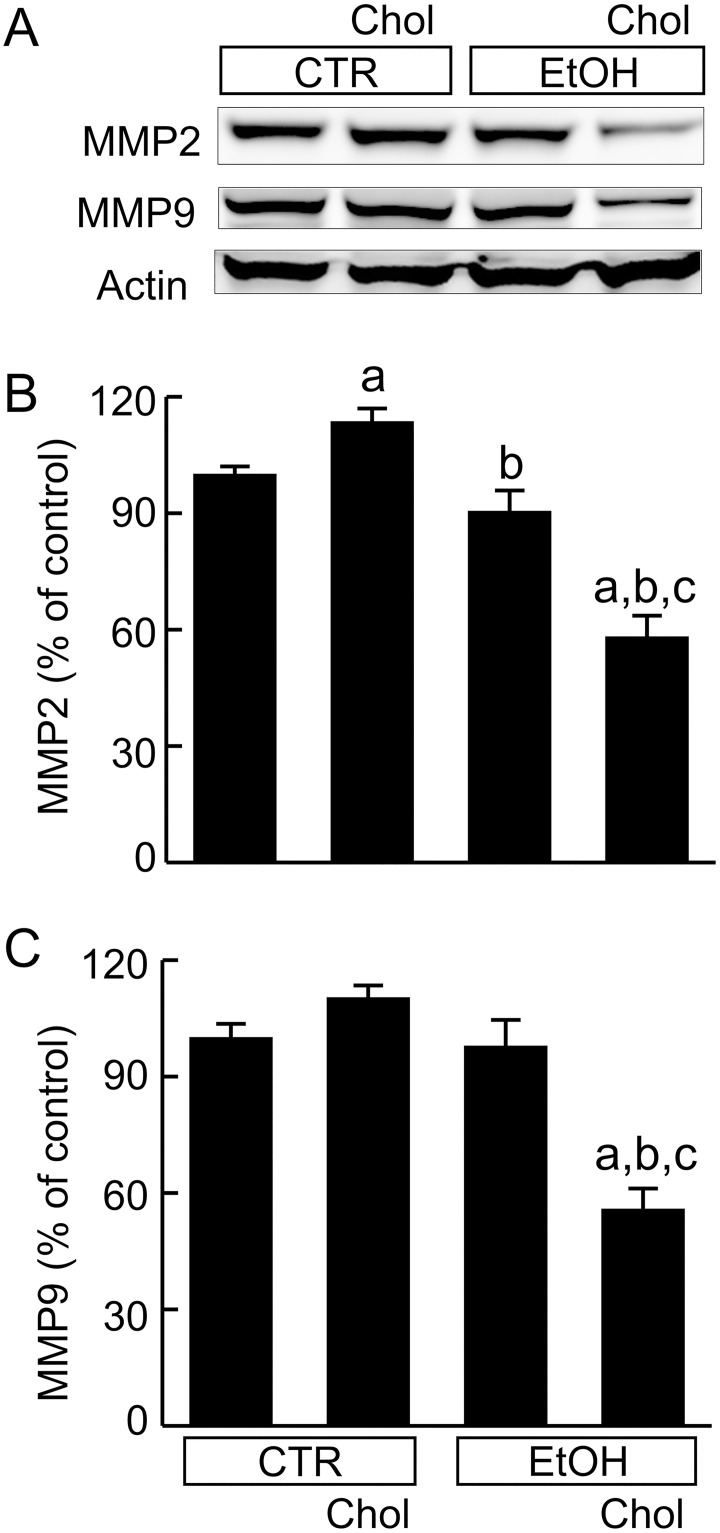
Combined ethanol and cholesterol feeding decreases metalloproteinase expression in the liver. Mice were fed CTR, Chol, EtOH and EtOH+Chol diets for 3 months. **A**, representative immunoblots of MMP-2, MMP-9 and actin. **B**, quantification of MMP-2 immunoblots by densitometry. **C**, quantification of MMP-9 immunoblots. Values are means ± S.E.M. **a**, p < 0.05 vs CTR; **b**, p < 0.05 vs Chol; **c**, p < 0.05 vs EtOH (n = 4 per group).

MMP degrade ECM. MMP-2 increased slightly (14%) by Chol feeding, was unchanged by EtOH, and decreased 42% by EtOH+Chol (Fig 7A and 7B). MMP-9 was not changed by Chol or EtOH feeding but decreased 44% by EtOH+Chol (Fig 7A and 7C).

### Combined ethanol and cholesterol consumption did not alter ethanol metabolic enzymes but increased oxidative and endoplasmic reticulum stresses

**Fig 8:**
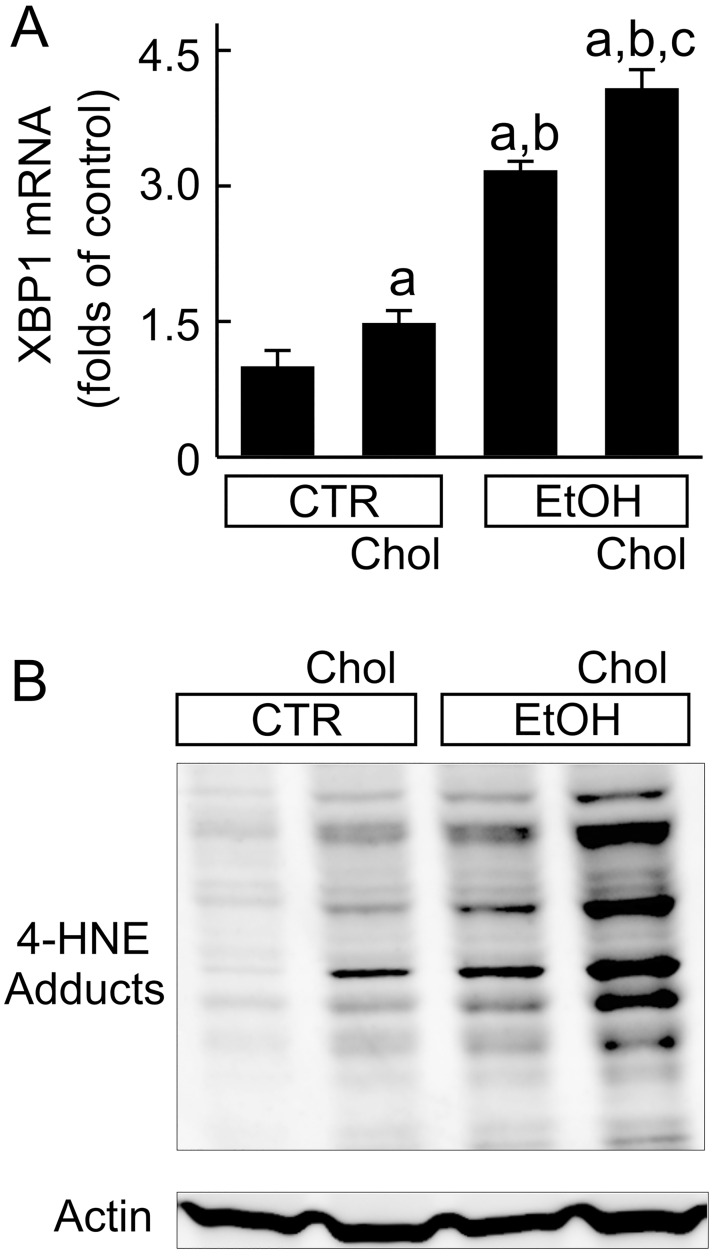
Combined ethanol and cholesterol feeding increases high mobility group box-1 mRNA and 4-hydroxynonenal adduct formation in the liver. Mice were fed CTR, Chol, EtOH and EtOH+Chol diets for 3 months. **A**, XBP-1 mRNA detected by RT-qPCR. Values are means ± S.E.M. **a**, p < 0.05 vs CTR; **b**, p < 0.05 vs Chol; **c**, p < 0.05 vs EtOH (n = 4 per group). **B**, representative immunoblots of 4-HNE adducts and actin (n = 4 per group).

Expression of ADH, Cyp2E1, and ALDH2, enzymes responsible for ethanol metabolism, were not different between EtOH+Chol feeding compared to Chol and EtOH (data not shown). Previous studies show that ethanol increases endoplasmic reticulum (ER) and oxidative stresses [29,30]. Expression of XBP-1 is an indicator of ER stress [31,32]. XBP-1 mRNA was increased slightly by Chol, more by EtOH and greatest by EtOH+Chol feeding (Fig 8A). 4-HNE is an indicator of oxidative stress. Multiple weak bands of 4-HNE adducts were detected in mice fed CTR (Fig 8B). 4-HNE adducts increased slightly after Chol, more after EtOH and greatest after EtOH+Chol (Fig 8B).

## Discussion

### Combined ethanol and high cholesterol consumption causes severe steatohepatitis and early fibrosis in mice

The pathogenesis of ALD is generally accepted to be a “multi-hit” process involving several risk factors [2,10,33]. In particular, nutritional factors appear to significantly modulate the pathogenesis and progression of alcohol-induced liver injury and fibrosis/cirrhosis [10,11]. Previous studies show that many patients with alcoholic hepatitis have some degree of malnutrition (low calorie and protein intake) [34,35]. High unsaturated fat and fish oil, high fructose intake, iron overload, and zinc deficiency are also reported to increase alcoholic liver injury [13,36–38]. Other studies suggest that high fat diets alter the intestinal microbiome and increase gut permeability, thus increasing endotoxemia and ALD development [37,39]. Obesity also increases the risk of ALD. Overall, nutritional factors and life styles most likely contribute to development of ALD by contributing to one or more of the “multi-hits” in ALD pathogenesis.

Western diets are high in fat and cholesterol. Therefore, excess ethanol consumption often occurs in combination with a high fat and high cholesterol diet. Recent studies show that high dietary cholesterol increases hepatic steatosis, inflammation, fibrosis and cancer in mice, but a high intake and long exposure time (1.5–5% for 25–55 weeks) are required [17,40–42]. Since non-alcoholic steatohepatitis (NASH) and alcoholic steatohepatitis (ASH) have similar pathological changes, we explored the combined effects of chronic ethanol consumption and high cholesterol intake. We showed that chronic cholesterol (0.5% for 12 weeks) alone caused steatosis, mild inflammation and cell death, whereas chronic ethanol alone induced slightly less steatosis than cholesterol but greater inflammation and cell death. Neither EtOH nor Chol diets alone caused observable liver fibrosis (Figs 1–5). By contrast after EtOH+Chol feeding, steatosis, inflammation and cell death all increased markedly over EtOH and Chol, and obvious liver fibrosis occurred (Figs 1–5). These data provide clear evidence that chronic ethanol and high cholesterol synergistically increase ALD development and severity.

### Mechanisms by which combined ethanol and high cholesterol consumption exacerbates steatohepatitis

How ethanol and cholesterol synergistically increase steatosis, cell death and inflammation, the three major components of steatohepatitis, remains unclear. Cholesterol does not appear to alter ethanol metabolism, since ADH, Cyp2E1 and ALDH2 expressions were not different in EtOH, Chol and EtOH+Chol groups (data not shown). Increased steatosis could result from increased *de novo* fatty acid synthesis, suppressed fatty acid degradation, and/or inhibited export of lipoproteins from the liver. *De novo* fatty acid synthesis is catalyzed by FAS. However, FAS expression decreased after EtOH+Chol feeding (Fig 2), indicating that hepatic steatosis was not due to increased *de novo* fatty acid synthesis. Alternatively, steatosis may be linked to suppressed fatty acid degradation in mitochondria. Entry of fatty acyl-CoA into mitochondria for β-oxidation is the rate limiting step in fatty acid degradation [23], Cpt1, an enzyme catalyzing an essential step in the transport of long chain fatty acyl-CoA into mitochondria, decreased markedly in EtOH+Chol mice (Fig 2). When mitochondrial uptake and oxidation of fatty acids is inhibited, fatty acyl-CoA is converted to triglyceride. This is consistent with our observed increase in hepatic triglycerides after EtOH+Chol feeding (Fig 2). Such inhibited fatty acid degradation most likely contributes to exacerbated steatosis by EtOH+Chol. Additionally, previous studies show that high dietary cholesterol and alcohol each impair very low density lipoprotein (VLDL) assembly and secretion [43–45]. Therefore, the combination of ethanol and high cholesterol consumption may also inhibit VLDL export, thus exacerbating accumulation of lipids in the liver.

Increased cell death by EtOH+Chol is possibly linked to higher ER and oxidative stresses. Previous studies show that ethanol increases ER stress and stimulates cholesterol trafficking and accumulation into mitochondria, which sensitizes mitochondria to oxidative stress [46]. Other studies also show that ethanol alone increases oxidative stress [47–50]. High cholesterol intake may further exacerbate these pathogenic processes. Indeed, we observed increases in XBP-1 mRNA and 4-HNE adducts in the liver after EtOH+Chol feeding (Fig 8A and 8B), consistent with increased ER and oxidative stresses by combined chronic ethanol and high cholesterol feeding. Oxidative stress is well known to damage macromolecules and organelles (*e*.*g*., DNA, proteins, cell membranes, mitochondria). Oxidative damage of membrane lipid components and proteins could compromise ion channels/transporters, enzymes and other plasma membrane activities. Mitochondria, the key bioenergetic organelle, are major producers as well as important targets of ROS [51,52]. Oxidative damage of cardiolipin, a mitochondrial phospholipid that is particularly rich in unsaturated fatty acids [53], suppresses the activity of cytochrome *c* oxidase of the electron transport chain [54]. Oxidative stress also causes onset of the mitochondrial permeability transition [55]. Failure of oxidative phosphorylation decreases ATP production, leading to necrotic cell death. Moreover, mitochondrial release of cytochrome *c* triggers apoptosis [55]. ER stress is also reported to cause apoptosis through mitochondria-dependent and -independent pathways [56]. Thus, combined ethanol and high cholesterol feeding increases cell death, likely by enhancing ER and oxidative stresses.

Increased ER and ROS stresses are also well-known contributors to inflammatory diseases. Activation of serine/threonine-protein kinase/endoribonuclease inositol-requiring enzyme 1 (IRE1) during ER stress recruits TNF receptor associated factor-2 (TRAF2) to the ER membrane to initiate inflammatory responses [57]. PRR activation is also reported to mediate ER stress-induced inflammation [57]. ROS stimulate formation of proinflammary cytokines/chemokines (*e*.*g*., TNFα, interleukin-1, CXC chemokine-10) and adhesion molecules [58,59]. Moreover, damage of hepatocytes after ROS and ER stress causes release of proinflammatory damage-associated molecular pattern molecules (DAMPs, *e*.*g*., mitochondrial DNA, HMGB1) [60,61]. These potent inflammatory mediators lead to infiltration of leukocytes, which produce more ROS, reactive nitrogen species, and proteases, further amplifying tissue damage.

Whether cholesterol increases inflammation remains controversial. Inflammation is an important component in ALD. Accumulation of free cholesterol in macrophages is a potent inducer of proinflammatory cytokine production [62]. In the liver, high dietary intake of cholesterol upregulates/activates proinflammatory signaling (e.g., NF-κB and TLR4) and increases inflammation in the liver [40,42,63]. High dietary cholesterol also increases production of mitochondrial ROS, which in turn activates the NLRP3 inflammasome [64]. In this study after EtOH+Chol feeding, HMGB-1 (a DAMP), TLR4 (a PRR), TNFα (a cytotoxic and inflammatory cytokine), and ICAM-1 (an adhesion molecule), all increased markedly in the liver, which was associated with overtly increased infiltration of leukocytes (Figs 1, 3 and 4). Our results support that cholesterol enhances inflammatory signaling after ethanol treatment, possibly by increasing ER and ROS stresses. However, a previous study shows that cholesterol suppresses inflammation in rat livers after ethanol exposure for a month [65]. The reason for this difference remains unclear, perhaps due to differences in species (mice vs rats) and/or duration of treatment. The effects of dietary cholesterol on low-density lipoprotein receptor and cholesterol synthesis are species-dependent, which could result in different cholesterol homeostasis and therefore affect inflammatory responses [66]. Moreover, the previous experiment was shorter (1 month) in duration in comparison to the current study (3 months). Thus, stimulation of the inflammatory response to ethanol by cholesterol may require a longer exposure time.

### Mechanisms by which combined ethanol and high cholesterol consumption causes liver fibrosis

Clinically ALD eventually progresses to cirrhosis, culminating liver failure and hepatic carcinoma [3–7]. However, induction of overt liver fibrosis by ethanol feeding in rodents has been difficult, which is an important barrier for development of antifibrotic therapies for ALD. A major difference between human and rodent diets is that normal rodent diets have minimal cholesterol, whereas human diets (especially in the Western countries) have high cholesterol content. It is possible that low cholesterol in rodent diets is the reason for the difficulty of inducing alcoholic fibrosis in rodents. Our study shows that chronic ethanol intake in combination with high cholesterol feeding leads to clearly apparent liver fibrosis with a pathological pattern consistent with alcoholic fibrosis (Fig 5). Thus, chronic ethanol plus high cholesterol feeding represents a new model for studying mechanisms and therapy for alcoholic fibrosis.

A previous study indicates that free cholesterol accumulates in cultured HSC, increasing TLR4 expression and sensitizing HSC to TGF-β [16]. These findings suggest that cholesterol may help to activate HSC *in vitro* and therefore stimulate fibrosis *in vivo*. Indeed, other recent studies show that cholesterol increases fibrosis in NASH in rodents, although this effect requires higher cholesterol (1.5–5%) feeding than used here and for longer periods of time (25–55 weeks) [16,17]. Previous studies also report that acetaldehyde, the reactive metabolite of ethanol, activates HSC in culture [67,68]. In the present study, we found that 0.5% cholesterol feeding alone only slightly increased Type 1 collagen, TGF-β1, α-SMA, and p-Smad 2/3 and did not result in frank fibrosis (Fig 5). BAMBI, a transmembrane TGF-β pseudoreceptor that silences TGF-β signaling [26], increased 60% in cholesterol-fed mice, which may have suppressed fibrosis (Fig 6). Furthermore, MMP2, which degrades ECM, was also slightly increased by cholesterol alone (Fig 7) to further delay fibrosis.

EtOH feeding alone caused somewhat greater pro-fibrotic responses compared to Chol alone (Figs 6 and 7), but outright fibrosis was still minimal (Fig 5). Although Chol and EtOH individually did not cause visible fibrosis, combined EtOH+Chol feeding caused definitive liver fibrosis (Fig 5). HSC activation, collagen formation and deposition, TGF-β and downstream TGF-β signaling all increased to much higher levels after EtOH+Chol feeding than after Chol or EtOH alone. Moreover, BAMBI and MMPs decreased 30–44% by EtOH+Chol (Figs 5–7). Together, these data indicated substantially increased profibrotic responses with decreased antifibrotic responses after EtOH+Chol feeding. Increased TGF-β is possibly linked to higher oxidative stress after EtOH+Chol feeding, since synthesis and activation of TGF-β, the key fibrogenetic cytokine, are stimulated by oxidative stress [69]. ER stress is also reported to induce fibrogenic responses in hepatic stellate cells [70]. Cholesterol homeostasis is regulated by sterol regulatory element-binding protein-2 (SREBP2) [16]. A recent study shows that high fat plus high cholesterol diet increases hepatic SREBP2 [16]. ER stress also stimulates expression of SREBPs [71]. Interestingly, the primary transcript of SREBP2 encodes miR33a, a micro RNA that enhances TGF-β/Smad signaling [16]. Therefore, increased SREBP2 may also increase miR33a formation and thus augment fibrosis. Indeed, miR33a markedly increased after EtOH+Chol feeding (Fig 6).

Taken together, this study demonstrated that combined ethanol and cholesterol consumption synergistically increased liver steatosis, injury, inflammation and fibrosis. These effects were most likely linked to oxidative and ER stresses, increased proinflammatory and profibrotic cytokine formation, and suppressed antifibrotic responses. Combined ethanol and cholesterol consumption mimics the Western diet style and provides a useful animal model for studying alcoholic steatohepatitis and liver fibrosis.
